# Vertebral Fractures in Ireland: A Sub-analysis of the DXA HIP Project

**DOI:** 10.1007/s00223-021-00868-7

**Published:** 2021-06-03

**Authors:** John J Carey, Lan Yang, E. Erjiang, Tingyan Wang, Kelly Gorham, Rebecca Egan, Attracta Brennan, Mary Dempsey, Catherine Armstrong, Fiona Heaney, Eva McCabe, Ming Yu

**Affiliations:** 1 School of Medicine, National University of Ireland Galway, Galway, Ireland; 2 Department of Rheumatology, Galway University Hospitals, Galway, Ireland; 3 School of Engineering, National University of Ireland Galway, Galway, Ireland; 4 Department of Industrial Engineering, Tsinghua University, Beijing, China; 5 Nuffield Department of Medicine, University of Oxford, Oxford, UK; 6 School of Computer Science, National University of Ireland Galway, Galway, Ireland

**Keywords:** Osteoporosis, DXA, Vertebral fractures, VFA

## Abstract

Osteoporosis is an important global health problem resulting in fragility fractures. The vertebrae are the commonest site of fracture resulting in extreme illness burden, and having the highest associated mortality. International studies show that vertebral fractures (VF) increase in prevalence with age, similarly in men and women, but differ across different regions of the world. Ireland has one of the highest rates of hip fracture in the world but data on vertebral fractures are limited. In this study we examined the prevalence of VF and associated major risk factors, using a sample of subjects who underwent vertebral fracture assessment (VFA) performed on 2 dual-energy X-ray absorptiometry (DXA) machines. A total of 1296 subjects aged 40 years and older had a valid VFA report and DXA information available, including 254 men and 1042 women. Subjects had a mean age of 70 years, 805 (62%) had prior fractures, mean spine T-score was − 1.4 and mean total hip T-scores was − 1.2, while mean FRAX scores were 15.4% and 4.8% for major osteoporotic fracture and hip fracture, respectively. Although 95 (7%) had a known VF prior to scanning, 283 (22%) patients had at least 1 VF on their scan: 161 had 1, 61 had 2, and 61 had 3 or more. The prevalence of VF increased with age from 11.5% in those aged 40–49 years to > 33% among those aged ≥ 80 years. Both men and women with VF had significantly lower BMD at each measured site, and significantly higher FRAX scores, *P* < 0.01. These data suggest VF are common in high risk populations, particularly older men and women with low BMD, previous fractures, and at high risk of fracture. Urgent attention is needed to examine effective ways to identify those at risk and to reduce the burden of VF.

## Introduction

Osteoporosis is one of the most common diseases worldwide resulting in millions of fragility fractures each year. 50% of women and 20–25% of men over the age of 50 years will experience an osteoporosis-related fracture, which are associated with significant morbidity, healthcare costs and increased mortality [1–3]. Although vertebral fractures (VF) represent the most frequently affected skeletal site, their impact is not fully appreciated compared with other skeletal sites [4, 5], due to their different risk factors, presentation and diagnostic criteria, and the use of vague or unclear terminology [6–11].

Epidemiologic studies of VF are heterogeneous, varying within and across different regions [6, 11–16]. However, their prevalence is broadly similar to hip fractures rising exponentially with age [6, 10, 13], in both men and women [6, 10–12, 17]. Their illness burden for patients and healthcare systems is also similar, but greater than forearm fractures [2–10, 18, 19]. Specific populations are at increased risk including those with rheumatic diseases [20–23], taking glucocorticoid therapy [24–28] or with previous fractures [13, 29–31].

Recent publications on the prevalence, incidence and impact of VF in Ireland highlight a paucity of studies [29, 32]. Reasons include under-representation, under-diagnosis and limited data from non-hospital settings [29, 32–34]. Reports suggest hospital length of stay is similar to hip fractures [33], while others show VF are common when imaging studies are systematically evaluated [29, 34]. Our experience suggests the prevalence and importance of VF are under-appreciated by patients, medical staff, healthcare managers and government.

Performance of vertebral fracture assessment (VFA) scans are recommended by the International Society for Clinical Densitometry (ISCD) for patients at risk [35]. In practice, these scans assess the prevalence amongst individuals [10, 13, 17, 34, 35] or the general population [17], or those of particular interest, such as rheumatoid arthritis [34] or fracture liaison service referrals [36]. In this study, we used available VFA scans from a sample of patients in the West of Ireland to gain a better appreciation of the prevalence of VF in men and women and to explore associations with conventional major risk factors, Bone Mineral Density (BMD) and FRAX scores.

## Methods

Data were extracted from 2 GE Lunar central DXA machines at a single centre as previously described [37], following approval from the institutional clinical research ethics committee (C.A. 2109). The committee granted a waiver of informed consent was granted for this study. These were saved, anonymised and subsequently available for analysis. The original cohort includes a sample of > 36,000 unique individuals with medical histories, medications and an array of DXA parameters. In this study, we include only subjects aged 40 years and older who had a VFA scan and available report from 1 of 2 GE Lunar machines at 1 centre over a 12-month period. A prior audit of > 7000 DXA referrals for our centre shows 69% are referred from primary care, 27% from hospital specialist clinics and 4% from inpatient services. Referrals are accepted per ISCD indications, or sent back to the referring clinician if more information is required or there is no appropriate indication [38]. Accepted referrals are then prioritised as ‘urgent’, ‘soon’, or ‘routine’ depending on the information provided and our impression of where the results are most likely to have the greatest clinical impact. For example, someone referred following a major fragility fracture and who is not on treatment would be ‘urgent’, while ‘screening’ for a postmenopausal woman aged 50 years with no other major risk factors would be considered routine. Waiting times for routine scans are currently 10 years, urgent scans are now < 3 months.

VFA scans are performed per ISCD indications (women aged > 70 years, men > 80 years, adults taking chronic glucocorticoid therapy, reporting height loss and reported but undocumented previous VF; the one difference being we do not apply the recommended T-score threshold) by 1 of 2 ISCD trained nurse specialists (RE and KG). All are read by a single ISCD trained and certified clinician experienced in reporting DXA and VFA scans (JJC) as per ISCD recommendations using the Genant semi-quantitative method for fracture ascertainment [35, 39, 40]. As part of our routine practice, all VFA scans are reviewed by our DXA nurse specialists at the time of scanning, and later by the reporting clinician. An outline of this process is as follows:

1. Trained DXA nurse specialists perform VFA scans and flag all patients with a known VF or an abnormal VFA scan with a suspected fracture for urgent review by the reporting clinician.
2. All VFA scans are reviewed and reported by the clinician routinely (other abnormalities are flagged for review, e.g., osteoarthritis, foreign body or other artefact).
3. Discrepancies, unusual or unclear scans are discussed at a weekly multi-disciplinary team audit with the lead clinician and 4 nurse specialists running the hospital’s DXA and Fracture Liaison Service (FLS). Agreement between our readers is currently >90% for moderate–severe fractures.
4. Additional imaging including X-rays, CT and MRI scans and reports are reviewed as necessary to verify anomalies or the presence of fractures. VFA report agreement is >70% for mild fractures.
5. Our FLS prioritises patients not on treatment with prevalent spine and hip fractures. Patients with multiple VF at the time of scanning are offered an immediate consult following their scan if a nurse specialist or clinician is available. Others are subsequently contacted as a priority, and offered an evaluation and treatment for osteoporosis or advised to follow-up urgently with their primary care doctor.
6. A copy of the DXA report highlighting the fractures and an FLS letter are sent to the patient’s doctor.

Demographic data were summarised as follows; age, gender, height, weight, BMI, primary and secondary indication for DXA scan, prior fracture, osteoporosis medication if prescribed, calcium and vitamin D supplementation, DXA T-scores, FRAX® variables, and FRAX® scores. DXA T-scores for all men and women are calculated using NHANES III reference data for white females [14]. We chose to classify all subjects as osteoporotic or not using ISCD criteria for older men and postmenopausal women, i.e., lowest T-score ≤ − 2.5 [14]. FRAX® scores were calculated by GE software FRAX® tool. VFA indications, scan technique and recommended reporting methods were performed as recommended by ISCD [35, 40]. Data for the entire cohort were summarised, compared by gender and by decade to enable comparison to publications from other countries [11, 12, 15–17]. The results of the VFA analysis are recorded as ‘fracture’ or ‘no fracture’, and also include the site for the first 2 fractures. If patients had > 2 VF sites, they were simply recorded as ‘multiple’. Details on fracture type and severity, or results of other imaging are not available in this dataset.

All analyses were performed using R statistical software, version 3.6.1. For categorical variables, we report subject count and percentage, and used Pearson’s Chi-squared test (if the number of subjects in each comparative group was ≥ 5) or Fisher’s exact test (if there were fewer than 5 subjects in a comparison group) to examine statistically significant differences between groups. For continuous variables, normality was assessing using Shapiro–Wilk’s test. We then calculated mean and standard deviation and used unpaired Student’s t tests to compare groups whose data were normally distributed, while for non-parametric data, we used data are expressed as medians and interquartile ranges, while Wilcoxon’s rank sum test examined for statistically significant differences between two groups, and Kruskal–Wallis test was used for comparing multiple groups. All significance tests carried out were two-sided, and *P* values < 0.05 were considered significant.

## Results

1296 subjects had a VFA scan report and DXA information available for analysis including 1042 women and 254 men. Details of our entire cohort has previously been published [37, 41], while a summary of the characteristics of this sub-group are shown in Table 1. The majority are female, with a mean age of 70 years, and a range of 40–94 years. Women were significantly lighter and smaller than men. The majority of men and women had a prior fracture and a substantial proportion were taking corticosteroids, had rheumatoid arthritis, a family history of osteoporosis or another illness or medication predisposing to osteoporosis (Table 1). All had at least 1 skeletal site available for analysis of BMD; spine: 1063 (82.0%), total hip and femoral neck: 1234 (95.2%) and 1/3 distal radius: 3 (0.2%). 1217 (93.9%) had GE Lunar FRAX scores for major osteoporotic fracture and hip fracture. 35% of patients were taking osteoporosis medication, 93.3% of whom were taking anti-resorptive medication. 6% were taking teriparatide, alone or in combination with an anti-resorptive, while 0.7% were taking other medication combinations.

**Table 1:** Summary of study subject characteristics

Variable	All (*N* = 1296)	Female (*N* = 1042)	Male (*N* = 254)	*P* value^a^
Age (year): mean ± SD	70.01 ± 10.51	70.25 ± 10.19	69.00 ± 11.69	0.117
Height (cm): mean ± SD	161.52 ± 9.27	159.07 ± 7.82	171.59 ± 7.84	< 0.001
Weight (kg): mean ± SD	71.55 ± 15.95	68.97 ± 14.47	82.14 ± 17.32	< 0.001
BMI (kg/m^2^): mean ± SD	27.33 ± 5.44	27.19 ± 5.46	27.92 ± 5.32	0.050
Prior fracture: *N* (%)	805 (62.1%)	675 (64.8%)	130 (51.2%)	< 0.001
Osteoporosis treatment: *N* (%)	460 (35.5%)	393 (37.7%)	67 (26.4%)	< 0.001
Corticosteroid use: *N* (%)	312 (24.1%)	192 (18.4%)	120 (47.2%)	< 0.001
Family history: *N* (%)	250 (19.3%)	235 (22.6%)	15 (5.9%)	< 0.001
Height loss: *N* (%)	119 (9.2%)	104 (10.0%)	15 (5.9%)	0.058
Rheumatoid arthritis: *N* (%)	187 (14.4%)	156 (15.0%)	31 (12.2%)	0.305
Secondary osteoporosis: *N* (%)	531 (41.0%)	423 (40.6%)	108 (42.5%)	0.626
Smoking: *N* (%)	120 (9.3%)	97 (9.3%)	23 (9.1%)	0.996
Spine T-score: mean ± SD	− 1.41 ± 1.52	− 1.65 ± 1.35	− 0.44 ± 1.81	< 0.001
Femur neck T-score: mean ± SD	− 1.53 ± 0.96	− 1.63 ± 0.89	− 1.09 ± 1.13	< 0.001
Total hip T-score: mean ± SD	− 1.21 ± 1.19	− 1.37 ± 1.09	− 0.58 ± 1.35	< 0.001
Lowest T-score ≤ − 2.5: *N* (%)	409 (31.6%)	363 (34.8%)	46 (18.1%)	< 0.001
FRAX MOF^b^ (%): mean ± SD	15.4 ± 8.5	16.8 ± 8.6	9.4 ± 4.9	< 0.001
FRAX hip (%): mean ± SD	4.8 ± 5.3	5.0 ± 5.5	3.7 ± 3.6	< 0.001

^a^Comparison between genders

^b^FRAX® (Ireland) 10-year probability of major osteoporotic fracture

409 (31.6%) of the 1296 subjects had a DXA classification (T-score ≤ − 2.5) of osteoporosis (Table 1). The prevalence of osteoporosis increased steadily with age, particularly for women, from 16.1% in those aged 40–49 years, to 41.3% amongst those 80 years and older (Table 2). Mean BMD was significantly lower for women than men, and the proportion of women classified as osteoporotic by DXA was almost twice that of men (Table 1). Women had higher mean fracture risk scores than men (Table 1). Although the 10-year risk of fracture (GE Lunar FRAX®) increased with age for both genders, women had higher scores at each age category (Table 2).

**Table 2:** Prevalence of osteoporosis (Lowest T-score ≤ − 2.5) and mean FRAX® major osteoporotic fracture risk by age and gender

		Age					*P* value^c^
		40–49	50–59	60–69	70–79	≥ 80	
All	Prevalence: N (%)	9 (15.4)	42 (26.9)	122 (30.7)	127 (29.4)	110 (42.5)	
Female	Prevalence: N (%)	5 (16.1)	36 (29.3)	111 (35.2)	118 (33.2)	93 (41.3)	0.003
Male	Prevalence: N (%)	3 (14.2)	6 (18.2)	11 (15.3)	9 (11.7)	17 (33.3)	0.043
*P* value^a^		1	0.292	0.003	< 0.001	0.189	

		Age					*P* value^d^
		40–49	50–59	60–69	70–79	≥ 80	
All	FRAX (± SD)	5.9 (3.4)	9.0 (5.8)	14.2 (7.1)	17.5 (8.0)	20.0 (9.4)	
Female	FRAX (± SD)	6.0 (3.8)	9.5 (6.0)	15.2 (7.1)	19.0 (7.7)	22.2 (9.0)	< 0.001
Male	FRAX (± SD)	5.8 (2.8)	6.8 (4.2)	9.3 (4.4)	10.7 (5.4)	10.9 (4.8)	< 0.001
*P* value^b^		0.725	0.003	< 0.001	< 0.001	< 0.001	

^a^Comparison of prevalence of osteoporosis between genders in each age group using Chi square or Fisher’s test

^b^Comparison of FRAX MOF between genders in each age group using Wilcoxon test

^c^Comparison of prevalence of osteoporosis between age groups in each gender group using Chi square or Fisher’s test

^d^Comparison of FRAX MOF between age groups in each gender group using Kruskal–Wallis test

805 patients (62.1%) had a prior fracture (Table 3). Hip fractures are more common in men, while wrist/forearm fractures are more common in women (both *P* < 0.001). The prevalence of spine, humerus, and other fractures is similar between both genders. Almost 1 in 3 patients have multiple (> 1) fractures. 95 (7.3%) patients had a known VF prior to scanning, 84 (88%) of whom had a visible reported fracture on their scan. An additional 199 (15.4%) patients had a VF visible on their VFA scan.

**Table 3:** Site of previous fracture of study subjects

	All (*N* = 805)^b^	Female (*N* = 675)	Male (*N* = 130)	*P* value^a^
Hip	59 (7.3%)	39 (5.8%)	20 (15.4%)	< 0.001
Humerus	39 (4.8%)	33 (4.9%)	6 (4.6%)	1.000
Spine	95 (11.8%)	74 (11.0%)	21 (16.2%)	0.126
Wrist/forearm	185 (23.0%)	145 (21.5%)	8 (6.2%)	< 0.001
Other	179 (22.2%)	147 (21.8%)	34 (26.2%)	0.327
2 or more sites	243 (30.2%)	204 (30.2%)	39 (30.0%)	1.000
Unknown site(s)	5 (0.6%)	3 (0.4%)	2 (1.5%)	0.186

^a^Comparison of site of previous fracture between genders using Pearson’s Chi-squared test (if the number of subjects in each comparative group is greater or equal to 5); otherwise, Fisher’s exact test was performed

^b^805 (62.1%) of 1296 study subjects had a prior fracture

**Fig. 1:**
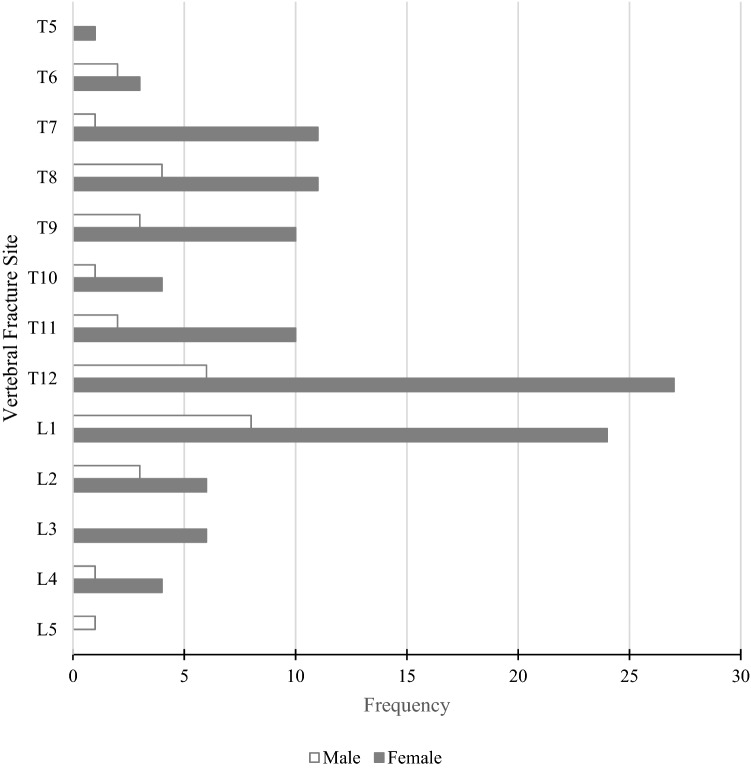
Vertebral fracture sites of study subjects with 1 fracture site

**Fig. 2:**
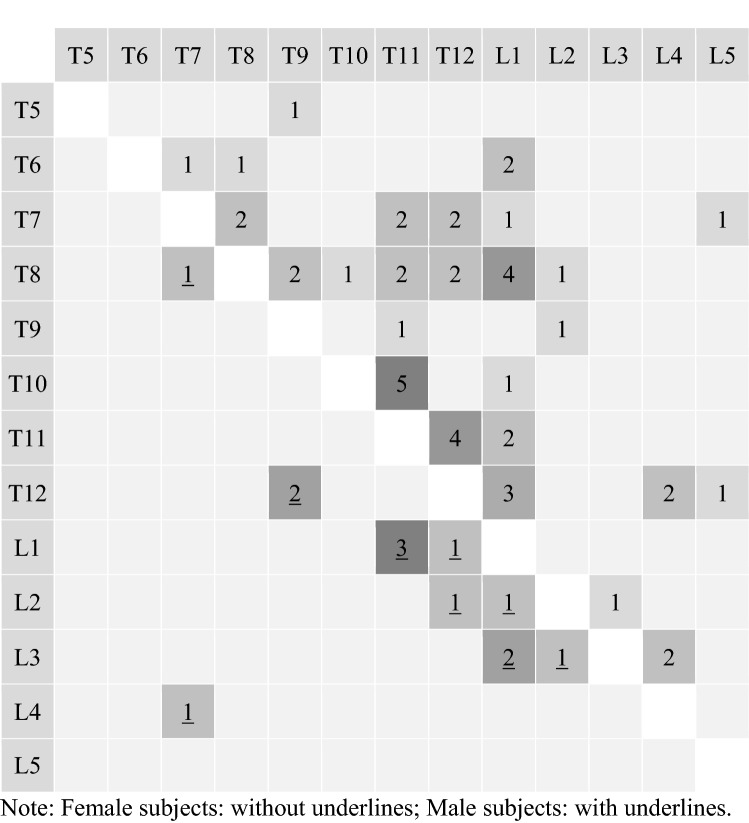
Heat map of vertebral fracture sites of study subjects with 2 fracture sites. Female subjects: without underlines; male subjects: with underlines

283 (21.8%) patients had at least 1 VF on their VFA scan, including 225 (21%) women and 61 (23%) of men. 152 had a single fracture, and a further 61 had 3 or more fractures. The sites for one fracture are shown in Fig. 1, while the sites and frequency of multiple fractures are shown in Fig. 2. The proportion of subjects with 1, 2 and 3 or more VF was similar across both genders (Women vs. Men: 11.2% vs. 12.8%, 4.5% vs. 4.9% and 4.5% vs. 4.9%, respectively). The prevalence of VF increased with age, a pattern which is significantly more striking in women (Table 4). Although men experienced an increase in VF with age, this trend was offset by the high proportion of men aged 40–49 years with VF.

**Table 4:** Prevalence of vertebral fracture by age and gender

Prevalence: % (*N*)	Age					*P* value^b^
	40–49	50–59	60–69	70–79	≥ 80	
All	11.5% (6)	16.0% (25)	16.9% (67)	22.9% (99)	33.2% (86)	
Female	3.2% (1)	16.3% (20)	16.3% (53)	22.3% (79)	34.1% (71)	< 0.001
Male	23.8% (5)	15.2% (5)	19.4% (14)	26.0% (20)	29.4% (15)	0.531
*P* value^a^	0.034	1	0.639	0.579	0.634	

^a^Comparison of prevalence of vertebral fracture between genders in each age group using Pearson’s Chi-squared test (if the number of subjects in each comparative group is greater or equal to 5); otherwise, Fisher’s exact test was performed

^b^Comparison of prevalence of vertebral fracture between age groups in each gender using Pearson’s Chi-squared test (if the number of subjects in each comparative group is greater or equal to 5); otherwise, Fisher’s exact test was performed

Table 5 summarises the association between the presence of VF, classification of osteoporosis, fracture risk and major risk factors for fracture. The majority of VF (57%) occurred in individuals with a T-score > − 2.5 (Table 5). However, subjects with a DXA classification as osteoporosis (lowest T-score ≤ − 2.5) were more likely to have VF compared to subjects without: 29.8% vs. 18.8% (*P* < 0.001). Not surprisingly, 10-year fracture risk scores were significantly higher amongst those with VF than those without (Table 5). People with VF were also older, lighter and more likely to have a prior fracture, height loss and to be on osteoporosis treatment. Interestingly, people with VF were also less likely to be taking corticosteroids or have other causes of osteoporosis.

**Table 5:** Comparison of subjects stratified by presence of vertebral fracture

Variable	All (*N* = 1296)	With vertebral fracture (*N* = 283)	Without vertebral fracture (*N* = 1013)	*P* value^a^
Age (year): mean ± SD	70.01 ± 10.51	72.88 ± 10.32	69.21 ± 10.42	< 0.001
Height (cm): mean ± SD	161.52 ± 9.27	160.39 ± 8.85	161.84 ± 9.36	0.016
Weight (kg): mean ± SD	71.55 ± 15.95	69.17 ± 15.37	72.22 ± 16.05	0.004
BMI (kg/m^2^): mean ± SD	27.33 ± 5.44	26.84 ± 5.36	27.47 ± 5.46	0.086
Prior fracture: *N* (%)	805 (62.1%)	240 (84.8%)	565 (55.8%)	< 0.001
Osteoporosis treatment: *N* (%)	460 (35.5%)	147 (51.9%)	313 (30.9%)	< 0.001
Corticosteroid use: *N* (%)	312 (24.1%)	45 (15.9%)	267 (26.4%)	< 0.001
Family history: *N* (%)	250 (19.3%)	53 (18.7%)	197 (19.5%)	0.853
Height loss: *N* (%)	119 (9.2%)	46 (16.3%)	73 (7.3%)	< 0.001
Rheumatoid arthritis: *N* (%)	187 (14.4%)	40 (14.1%)	147(14.5%)	0.949
Secondary osteoporosis: *N* (%)	531 (41.0%)	91 (32.2%)	440(43.4%)	< 0.001
Smoking: *N* (%)	120 (9.3%)	33 (11.6%)	87 (8.6%)	0.144
Spine T-score: mean ± SD	− 1.41 ± 1.52	− 1.88 ± 1.51	− 1.31 ± 1.51	< 0.001
Femur neck T-score: mean ± SD	− 1.53 ± 0.96	− 1.89 ± 0.94	− 1.43 ± 0.95	< 0.001
Total hip T-score: mean ± SD	− 1.21 ± 1.19	− 1.72 ± 1.13	− 1.08 ± 1.17	< 0.001
Lowest T-score ≤ − 2.5: *N* (%)	409 (31.6%)	122 (43.1%)	287 (28.3%)	< 0.001
FRAX® MOF^b^ (%): mean ± SD	15.4 ± 8.5	19.3 ± 9.3	14.3 ± 8.0	< 0.001
FRAX® Hip (%): mean ± SD	4.8 ± 5.3	7.0 ± 6.9	4.2 ± 4.6	< 0.001

^a^Comparison between with and without VF

^b^FRAX® 10-year probability of major osteoporotic fracture

## Discussion

In this study examining lateral DXA spine scans, we found VF are common in people with or at high risk for fracture or with established osteoporosis. The prevalence of VF increased with age, particularly in women. The prevalence of VF in this population was actually 3 times higher than their known VF prevalence prior to their VFA scan, many of whom had 2 or more VF. Although the majority of the study’s cohort did not meet the DXA threshold to be classified as osteoporotic, the presence of VF was significantly associated with lower BMD and higher fracture risk scores. These data have important implications for considering VFA scans in Irish adults undergoing DXA.

VF represents a major problem in osteoporosis care, and the commonest site of injury [2, 6, 9, 10, 13, 17]. Clinical fractures have a similar impact on quality of life, morbidity and mortality to hip fractures [4, 9, 19]. Unlike other fractures, the presence and importance of VF are greatly under-appreciated [6–8, 10, 12, 13, 29]. General population studies from Europe, China, Latin and North America show VF prevalence increases with age in both men and women, irrespective of which criteria are used [6, 11, 12, 15–17]. VF are more prevalent in our study than general population studies, because a VFA scan was performed on the basis of risk factors [14, 35]. Thus, a large proportion already have osteoporosis and fractures, are older, have rheumatoid arthritis, or are taking corticosteroid medications, all of which represent significant risk factors [1, 10, 13, 34].

Rheumatic diseases are associated with a higher risk of osteoporotic fracture, particularly rheumatoid arthritis and ankylosing spondylitis [20–23, 34, 42]. The risk of VF in ankylosing spondylitis is almost threefold higher [20, 22] which is similar to rheumatoid arthritis [21, 42]. 14% of our study population have rheumatoid (14%), which is much greater than the population prevalence. Glucocorticoids are commonly used to treat rheumatic diseases and increase the risk of osteoporotic fracture, particularly with higher doses [24–28, 43]. Clinical trials of glucocorticoid-induced osteoporosis show a strikingly high prevalence of osteoporotic fracture even in populations with normal or almost normal BMD [25–28, 43]. The prevalence of VF at baseline ranges from 10 to 36% in those with normal BMD [26, 28, 43] and 13% to 37% in those with low BMD [25, 28]. Patients with a prior fracture are at much greater risk of fracture [29–31], and although osteoporosis treatment is effective, fractures can still occur [25–28, 43]. In our study, patients with VF were more likely to have a prior fracture (85% vs. 56%), have osteoporosis by DXA criteria (43% vs. 28%), and be taking osteoporosis treatment (52% vs. 31%).

In this study, we found the prevalence of fractures was threefold higher than the reported prevalence prior to scanning, perhaps reflecting the nature of these fractures, where only around 1 in 3 present with clinical symptoms [6, 10, 13]. Unfortunately, we do not have clinical information on the presence or absence of back pain or other symptoms in this dataset. These results suggest the spine is the commonest site of fracture in this population, which is under-represented in published data for our country [29, 33]. 70% of people in this study did not have a DXA T-score below the osteoporosis threshold. This is in line with others [17], and a well described epidemiologic phenomenon [44]. Although some believe fractures in people whose BMD is not below the fracture threshold do not need treatment, a diagnosis of osteoporosis can be made and treatment offered in the presence of a major osteoporotic fracture and the absence of major trauma or other explanations [14, 32, 45]. Others have shown the risk of subsequent fracture is significantly higher in patients with low BMD and prevalent fractures than those with low BMD alone [30, 31].

Our study has important strengths and limitations. Prior studies in European populations have not included subjects from Ireland [11], and gaps remain in our understanding of the epidemiology and utility and applicability of diagnostic and risk tools for our populations [32]. Our study includes more than 1000 adults referred for VFA scanning, all of whom met ISCD indications for a scan. Many, therefore, had established osteoporosis, a prior fracture or were at high risk for fracture, so our results are not generalizable to the Irish population, such as those described by others [11, 12, 15–17], or our whole cohort [37].

The sample contains few men compared to some larger studies and likely reflects selection bias, particularly those 40–49 years of age. A prior study of a cohort of rheumatoid arthritis (RA) patients reported VF prevalence using this technology [34], but there are limited data on other populations and more than 85% of our population did not have RA. Clinicians and nurses were not blinded to the patients’ diagnoses and DXA results which could have resulted in ascertainment bias. Fracture type and grade were not available, nor the dose and duration of corticosteroids, which are further important limitations. We are conservative in diagnosing mild fractures as per ISCD recommendations [35, 40], and may have over or under-estimated the true prevalence of VF using this approach. Reassuringly agreement between our department VFA readings and other radiology reports is 88% for those with a known VF. Others have shown the prevalence changes substantially depending on which imaging method, analysis and criteria are employed [8, 11, 12]. We have also shown that a formal process to review available images results in a significant increase in fracture diagnosis [29, 34].

In our experience the presence and importance of VF for patients is greatly under-appreciated. Patients are often dismissed by health professionals when presenting with milder symptoms before they finally obtain imaging to confirm their diagnosis. In practice when the results could change patient management and there is doubt about the diagnosis, additional imaging should be sought or reviewed. Our results show that in persons deemed at increased risk for vertebral fracture, VFA scanning significantly increased the diagnosis of vertebral fractures, the presence of which is under-appreciated. This could have significant implications for the diagnosis and management of patients with osteoporosis at significant risk of subsequent fracture.

## Summary and Conclusions

In this study, we show that VF are common in high risk Irish adults, many of whom are unaware of their presence or who do not meet a DXA threshold for osteoporosis. Careful consideration should be given to performing VFA scans in high risk populations such as this.
